# Asymmetric polarization by vaccination status identification during the COVID-19 pandemic

**DOI:** 10.1371/journal.pone.0311962

**Published:** 2024-11-26

**Authors:** Sebastian Jungkunz

**Affiliations:** 1 Institute of Political Science and Sociology, University of Bonn, Bonn, Germany; 2 Institute of Political Science, University of Bamberg, Bamberg, Germany; Yeditepe University, TÜRKIYE

## Abstract

COVID-19 prevention measures and vaccine policies have led to substantial polarization across the world. I investigate whether and how vaccination status and vaccination status identification affect the sympathy and prejudice for vaccinated and unvaccinated individuals. Drawing on a preregistered vignette survey experiment in a large representative sample from Germany (n = 6,100) in December 2021, I show that prejudice was greater among the vaccinated towards the unvaccinated than vice versa. Furthermore, I find that differences in sympathy ratings are strongly subject to vaccination status identification. If individuals do not identify with their vaccination status, there are no differences in the evaluation of the in- and outgroups. Stronger vaccination status identification is, however, associated with greater prejudice among the vaccinated towards the unvaccinated but not for the unvaccinated towards the vaccinated. The results therefore show a stronger polarization on the side of the vaccinated that increases with the identification of one’s vaccination status.

## Introduction

COVID-19 prevention measures and vaccine policies have led to substantial polarization across the world [1]. Vaccination status, in particular, has become a dividing line in societies. Whereas vaccinated citizens considered vaccination as a supposedly moral duty to combat the spread of the coronavirus, the unvaccinated have often experienced substantial discrimination, e.g. through the restriction of their civil liberties and the access to various areas of social life, public transport, education, or work [2–5]. This has often been accompanied by public and political discourse that stigmatized the unvaccinated through blaming and scapegoating (“pandemic of the unvaccinated”) and substantial moral grandstanding by representatives of politics and the media, but also the public [6–8]. These developments therefore likely increased discrimination, either due to moral righteousness among the vaccinated or due to feelings of exclusion from the mainstream society among the unvaccinated (see also further [9–11]).

Previous research has shown that anger plays a substantial role in shaping social and political polarization around COVID-19 related issues [12]. Online discourse was also highly polarized which resulted in echo-chamber effects among both groups [13–16]. The degree to which individuals identify with their vaccination status has further been shown to be related to perceptions of discriminatory public discourse, everyday discrimination, and ostracism, but also greater preferences for ingroups [17].

I extend this research by investigating whether and how vaccination status and vaccination status identification affect the sympathy and prejudice for the respective outgroup. To do so, I conducted a survey in Germany between December 10 and 19, 2021 (n = 6,100) asking respondents about their COVID-19 vaccination status and how strongly they identify with their status. The survey contained a preregistered vignette survey experiment in which respondents were requested to evaluate certain social groups that varied according to their vaccination status, nationality, and party identification.

I preregistered two main hypotheses. First, I expected that vaccinated citizens perceive unvaccinated citizens more negatively than vaccinated ones (H1a). However, I also assumed that the relationship could be reversed so that unvaccinated citizens perceive vaccinated citizens more negatively than unvaccinated ones (H1b). Secondly, I predicted that stronger identification with one’s vaccination status is associated with stronger negative evaluation against the outgroup (H2). In sum, I find strong evidence for H1a and somewhat weaker support for H1b. Most importantly, the results show that higher vaccination status identification is associated with a substantially higher discrimination against the unvaccinated by the vaccinated. Unvaccinated citizens, in turn, show no stronger signs of prejudce against the vaccinated with higher levels of vaccination status identification. These findings thus indicate an asymmetrical polarization by vaccination status identification.

## Method

### Sampling and participants

The study was conducted using a recruited Bilendi & respondi online access panel in Germany (n = 6,100) between December 10 and 19, 2021. The average response rate in the access panel is 40%. Data and replication materials are available in the author’s Harvard Dataverse [18]. Germany is a particularly interesting case, as politicians strongly considered the introduction of compulsory vaccination against COVID-19 for the general public and introduced compulsory vaccination for healthcare workers.

The sample is representative in terms of age, sex, education, and regional distribution across east and west Germany. The target population were individuals of the public age 18 to 65. Panel recruiting and membership is voluntary and based on a double-opt-in registration process. Participants provided informed consent in written form to participate in the study by clicking a button on the web interface before being able to start the survey. The study contained a preregistered vignette survey experiment which can be accessed at https://aspredicted.org/blind.php?x=3BP_5SW. The sample was 50% female, had a mean age of 43 years, and 48.0% had a school leaving degree that grants university access (*Fachhochschulreife* or *Abitur*). 82.5% had received (at least) two doses of a COVID-19 vaccine (n = 5,032), 3.9% had received one dose (n = 237), 11.6% had received no dose (n = 706), and 2.0% indicated “don’t know” or did not want to answer the question (n = 125). This is comparable to official statistics which reported 61.5 million citizens that were vaccinated at least once by December 10, 2021. This translates into a share of 75.4% among the total population (including children) and 90.5% among the adult population [19, 20].

Summary statistics and a comparison with official census statistics can be found in S2 and S3 Tables in S1 Appendix. The average response time was 14.6 minutes. All model results are unaffected by the exclusion of speeders (fastest 3%, response time <343ms) and laggers (response time >10,000ms), or respondents that failed attention checks (unrelated to this study, see Appendix C in S1 Appendix).

### Operationalization

#### Dependent variable

The main dependent variable was assessed in a pre-registered vignette survey experiment consisting of 16 conditions (2x8) that were included in a between-subjects design. In the experiment, respondents were asked to evaluate a fictitious person with a randomized set of characteristics in terms of nationality or party identification (see Table 1 for detailed description). The person was then described as [vaccinated against COVID-19/does not want to get vaccinated against COVID-19]. After the treatment, respondents evaluated the sympathy of the person on a slider from -50 (very negative) to 0 (neutral) and +50 (very positive). Sympathy ratings served as dependent variable in all regression models. Further information about the vignette design is provided in Appendix A in S1 Appendix.

**Table 1:** Question wording of vignette.

Items	Question wording
Vignette	Imagine a person that [vaccination status] against COVID-19 and [nationality or party identity]: How likeable would you rate this person?
Vaccination status treatment	is vaccinated/does not want to get vaccinated
Nationality/Partisan Treatment	has German nationality/has Turkish nationality/identifies with the party platform of the CDU/identifies with the party platform of the SPD/identifies with the party platform of the Greens/identifies with the party platform of the FDP/identifies with the party platform of the Left Party/identifies with the party platform of the AfD

#### Vaccination status

Vaccination status was assessed by asking whether respondents are vaccinated against COVID-19. Response options included “Yes, fully”, “Yes, partly”, “No”, and “Don’t know/do not want to answer”. I combined fully and partly vaccinated respondents and removed respondents that did not specify a vaccination status (n = 125).

#### Identification with vaccination status

Identification with vaccination status was measured as how strongly respondents feel connected to people who have been vaccinated against COVID-19 (filter for vaccinated respondents) or do not want to be vaccinated against COVID-19 (filter for unvaccinated respondents). Responses were given on a seven-point Likert scale from 1 (not at all) to 7 (very strongly).

#### Control variables

Regression models for Fig 2 (and S1 Fig in S1 Appendix) also controlled for education, sex, age, and living in east or west Germany (see S1 Appendix for details). Education was based on school-leaving qualification certificates. I recoded education as “high” if respondents indicated they obtained a degree that grants college access (*Fachhochschulreife*, *Abitur*) and “low” for other degrees. I further dichotomized region into respondents living in eastern Germany or western Germany.

## Results

To analyze the vignette survey experiment, I ran linear regression models predicting sympathy values for treatment groups (German nationals, Turkish nationals, mainstream and AfD party supporters, vaccinated, and unvaccinated persons) by German citizenship (for German nationals and Turkish nationals treatment), vote choice (for mainstream parties and AfD treatment), and vaccination status (for vaccinated and unvaccinated treatment).

**Fig 1:**
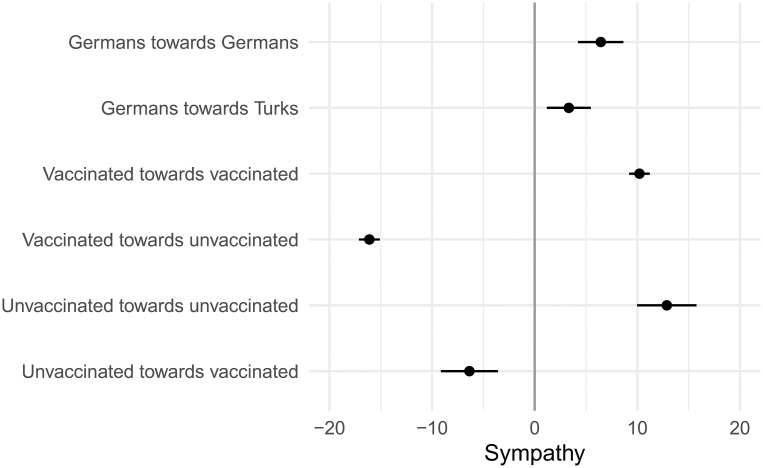
Predicted sympathy values with 95% confidence intervals by nationality and vaccination status. Sympathy ranges from 50 (low) to +50 (high). For interpretation: “Mainstream party voters towards AfD” refers to the sympathy of voters of mainstream parties (CDU/CSU, SPD, Greens, and FDP) towards AfD supporters. “Vaccinated towards unvaccinated” represents the sympathy of the vaccinated towards unvaccinated persons.

Fig 1 reports the predicted mean values from OLS models by treatment groups. Models including control variables are basically identical and are reported in S1 Fig in S1 Appendix. To benchmark the degree of sympathy and prejudice between the vaccinated and the unvaccinated, I first present the evaluation of Germans and Turks by German citizens. Overall, both are rated slightly favorably with Germans (mean = 6.438, 95%-CI [4.231; 8.646]) somewhat but not significantly higher than Turks (mean = 3.325, 95%-CI [1.185; 5.466]).

The third row then shows the sympathy ratings of mainstream party voters (CDU/CSU, SPD, Greens, FDP) towards AfD supporters (mean = -25.595, 95%-CI [-27.929; -23.260]) which are undoubtedly the most negative ratings out of all comparisons presented here. In turn, AfD voters rate mainstream party supporters much less negatively (mean = -3.862, 95%-CI [-6.288; -1.435]). These values are comparable to other findings prior to and at the onset of the pandemic [21–23], which also documented the most severe divides among various social groups among partisans, particularly for mainstream party voters disliking the far right [24].

The results in the fifth and seventh row show that both the vaccinated (mean = 10.209, 95%-CI [9.208; 11.210]) and the unvaccinated (mean = 12.874, 95%-CI [9.985; 15.764]) evaluate their own group significantly higher than German nationals, but they share about the same sympathy rating for their own ingroup. Most importantly however, I find that the vaccinated discriminate significantly more strongly against the unvaccinated (mean = -16.112, 95%-CI [-17.133; -15.092]) than the unvaccinated discriminating against the vaccinated (mean = -6.364, 95%-CI [-9.146; -3.582]). To put these numbers in perspective, the vaccinated dislike the unvaccinated on average about half as much as maintream party voters dislike AfD supporters. While this confirms both hypotheses H1a and H1b, the degree of discrimination is substantially weaker among the unvaccinated towards the vaccinated.

**Fig 2:**
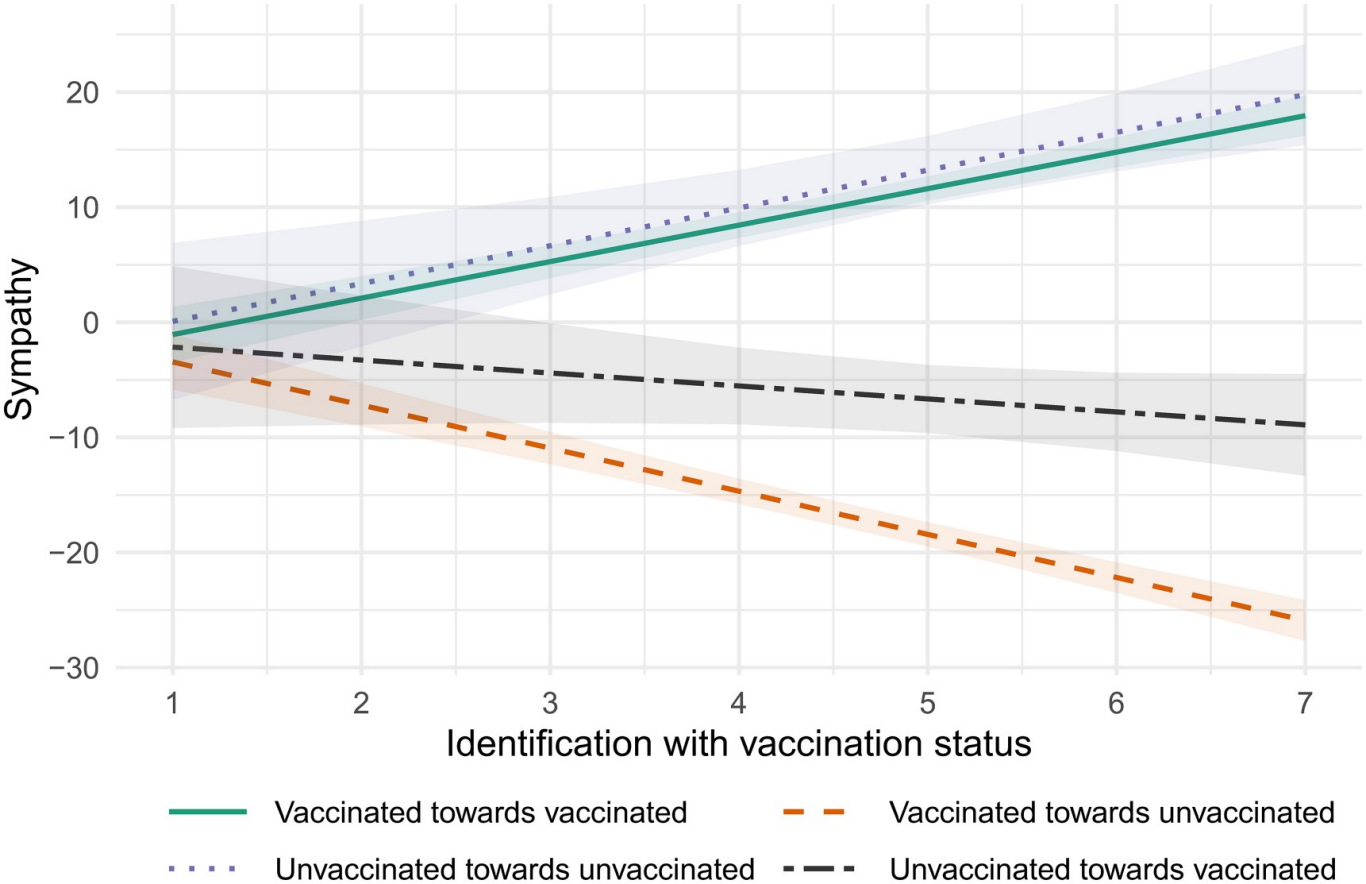
Predicted sympathy values with 95% confidence intervals by vaccination status identification. Sympathy ranges from 50 (low) to +50 (high), identification with vaccination status from 1 (none) to 7 (strong). For interpretation: “Vaccinated towards unvaccinated” represents the sympathy of the vaccinated towards unvaccinated persons.

Fig 2 then shows the predicted sympathy values by degrees of vaccination status identification. I again ran linear regression models predicting sympathy values for treatment groups (vaccinated and unvaccinated persons) by vaccination status and degree of vaccination status identification. Models reported in the text additionally control for age, sex, education, and living in eastern or western Germany. Missing values were excluded through listwise deletion.

In general, the unvaccinated (mean = 4.953, SD = 1.886) identify slightly stronger with their vaccination status than the vaccinated (mean = 4.622, SD = 1.721), although not substantially (t(808.141) = 4.2674, p<0.001, d = 0.190). There are no differences in sympathy ratings between in- or outgroups if citizens do not identify with their vaccination status. The findings first show that ingroup sympathy increases substantially and significantly for the vaccinated (from: mean = -1.066, 95%-CI [-3.469; 1.336], to: =17.952, 95%-CI [16.224; 19.681]) and the unvaccinated (from: mean = 0.081, 95%-CI [-6.724; 6.885], to: =19.788, 95%-CI [15.430; 24.146]) as vaccination status identification increases from no identification to strong identification. There are no differences between both groups.

However, vaccination status identification shows significantly and substantially different patterns for vaccinated and unvaccinated individuals on the evaluation of the opposing group. Stronger identification with the vaccination status is associated with a substantial decrease in sympathy for the unvaccinated among vaccinated respondents (from: mean = -3.445, 95%-CI [-5.838; -1.052], to: = -25.916, 95%-CI [-27.682; -24.150]). To put this in perspective, vaccinated respondents with strong vaccination status identification dislike the unvaccinated as much as mainstream party voters dislike AfD supportes (see Fig 1). In turn, stronger vaccination status identification is not associated with significantly higher prejudice against the vaccinated among unvaccinated respondents (from: mean = -2.154, 95%-CI [-9.165; 4.857], to: = -8.911, 95%-CI [-13.323; -4.500]). This confirms hypothesis H2 but only for vaccinated individuals.

Finally, I tested the robustness of the findings using additional robustness checks using attention checks and removing speeders and laggers. Overall, the findings in S2–S7 Figs in S1 Appendix are basically identical to the results presented in the main text.

## Discussion and conclusion

This study investigated the role of vaccination status identification during the COVID-19 pandemic on the polarization of vaccinated and unvaccinated individuals. On the one hand, it was assumed that prevention measures, vaccine policies, and the portrayal of the unvaccinated in the media were likely to lead to greater prejudice and stigmatization among the vaccinated. On the other hand, feelings of exclusion from the mainstream society may have affected the unvaccinated as well.

The results show that prejudice was greater among the vaccinated towards the unvaccinated than vice versa. This confirms similar findings in other countries [1]. Building on previous research that reports much stronger vaccination status identification among vaccinated individuals [25], I find, however, that differences in sympathy ratings are strongly subject to vaccination status identification. If individuals do not identify with their vaccination status, there are no differences in the evaluation of the in- and outgroups. Stronger vaccination status identification is associated with greater prejudice among the vaccinated towards the unvaccinated but not for the unvaccinated towards the vaccinated.

These findings are consistent with numerous studies from other countries across the world using surveys and experimental methods that now document robust evidence for greater discrimination against unvaccinated citizens by the vaccinated than vice versa [1, 7, 25–28]. COVID-19 vaccination status therefore served as a social identity: higher identification leads to stronger affective polarization and discrimination against respective vaccination status outgroups, but only among the vaccinated (see further [29, 30]).

To explain the asymmetry of this relationship, Schieferdecker and colleagues discuss three possibilities with regards to the acceptance of COVID-19 containment measures [31]: majority-minority effects, stereotyping, and the nature of issue positions. First, supporters of the measures viewed themselves as aligned with the broad public consensus and holding a morally urgent stance. This majority status thus fostered a stronger group identity and vilification of the minority opponents. Opponents, being a minority, felt alienated and less cohesive. Secondly, opponents were often pictured by politicians and the media as a radical, fringe group pursuing selfish interests, influenced by conspiracy theories, and linked to right-wing extremists. These negative stereotypes made it easier for supporters to identify with their group while alienating moderate opponents from their own group. Finally, supporters’ positions were portrayed as morally imperative (protecting lives), while opponents’ stances (protecting constitutional freedoms and the economy) appeared more abstract and self-serving, enhancing the polarization and making it harder for moderate opponents to identify with their own group. Although testing these claims is beyond the scope of this paper, it seems plausible that they may apply in the case of discrimination by vaccination status as well, as support for containment measures and vaccination against COVID-19 are strongly related [32, 33].

These results are also in line with other work that documents unintended negative consequences of COVID-19 prevention measures and vaccine policies [2, 34]. Bor et al. have shown that the moralization of COVID-19 compliance measures, e.g. due to paternalism, nudging, and judgementalism, increases the tendency to condemn and shame others ([6], see also [1, 2, 35–37]), which may also undermine general trust in science. In the U.S., this resulted, for instance, in a substantial decline among Republicans to consider vaccination against measles, mumps and rubella as required to attend public schools [38–40]. However, trust in health authorities increased if communication was transparent, even about negative effects of vaccines [41]. For credible science communication during crises in which policy-makers are faced with difficult trade-off situations, decisions and (protective) measures should therefore be based on reliable empirical data on the actual health risks involved, followed by transparent and open communication. Finally, policy-makers should strongly consider unintended negative consequences of future public health measures.

## Supporting information

S1 Appendix (PDF)
