# Supplementary material for: Asymmetric polarization by vaccination status identification during the COVID-19 pandemic
Source: PLoS One. 2024 Nov 26;19(11):e0311962. doi: 10.1371/journal.pone.0311962 (PMC11593748; doi:10.1371/journal.pone.0311962)
Supplement: S1 Appendix — (PDF) [file pone.0311962.s001.pdf]

Supplementary Material for "Asymmetric polarization by vaccination status identification during the COVID-19 pandemic"

A Vignette Experiment: Description and Treatment Assignment Checks

All respondents were presented with a vignette including one fully randomized treatment specification from Table S1. A cross-check for random assignment of vignettes along with the number of respondents per treatment is provided in Table S4. After the vignette respondents were presented a slider with which they could indicate their sympathy from very strong antipathy (-50) to very strong sympathy (+50).

Table S1: Question Wording of Vignettes

**Combination**	**Question wording**
Vaccinated & German nationality	Imagine a person that is vaccinated against COVID-19 and has German nationality
Vaccinated & Turkish nationality	Imagine a person that is vaccinated against COVID-19 and has Turkish nationality
Vaccinated & CDU	Imagine a person that is vaccinated against COVID-19 and identifies with the party platform of the CDU
Vaccinated & SPD	Imagine a person that is vaccinated against COVID-19 and identifies with the party platform of the SPD
Vaccinated & Greens	Imagine a person that is vaccinated against COVID-19 and identifies with the party platform of the Greens
Vaccinated & FDP	Imagine a person that is vaccinated against COVID-19 and identifies with the party platform of the FDP
Vaccinated & Left Party	Imagine a person that is vaccinated and identifies with the party platform of the Left Party
Vaccinated & AfD	Imagine a person that is vaccinated against COVID-19 and identifies with the party platform of the AfD
Unvaccinated & German nationality	Imagine a person that does not want to get vaccinated against COVID-19 and has German nationality
Unvaccinated & Turkish nationality	Imagine a person that does not want to get vaccinated against COVID-19 and has Turkish nationality
Unvaccinated & CDU	Imagine a person that does not want to get vaccinated against COVID-19 and identifies with the party platform of the CDU
Unvaccinated & SPD	Imagine a person that does not want to get vaccinated against COVID-19 and identifies with the party platform of the SPD
Unvaccinated & Greens	Imagine a person that does not want to get vaccinated against COVID-19 and identifies with the party platform of the Greens
Unvaccinated & FDP	Imagine a person that does not want to get vaccinated against COVID-19 and identifies with the party platform of the FDP
Unvaccinated & Left Party	Imagine a person that does not want to get vaccinated and identifies with the party platform of the Left Party
Unvaccinated & AfD	Imagine a person that does not want to get vaccinated against COVID-19 and identifies with the party platform of the AfD

For the calculation of our models we combined the treatments about nationality and partisanship in the models based on vaccination status.

B Tables

Table S2: Summary statistics

	N	Mean	SD	Min	Max
Female	6,099	0.49		0	1
Age	6,100	43.02	13.57	18	65
Education: high	6,072	0.48		0	1
East Germany	6,100	0.17		0	1
Thermometer	6,096	−2.14	29.40	−50	50
Vaccinated	5,975	0.88		0	1
Vaccination status identification	5,638	4.66	1.74	1	7

Table S3: Sample comparison with German Microcensus

	**Microcensus**	**Sample**
Female	50.5	49.3
Education: (Fach-)Abitur	41.0	48.0
East Germany	15.6	17.1
Age: 20-34	30.4	30.5
Age: 35-54	43.9	44.2
Age: 55-64	25.7	25.3

Official census statistics are based on the 2021 German Microcensus. Education statistics are based on age range 25 to 59 (Statistisches Bundesamt (Destatis) et al. 2021). Proportions of age groups are based on population aged 20 to 64. Percentages might not add up to 100 percent due to rounding.

Table S4: Balance tests for Treatment Assignment by Socio-Demographics

Treatment	Education	Female	Age	East German	Vaccinated	n
German & vaccinated	0.471	0.479	43.234	0.192	0.878	385
German & unvaccinated	0.522	0.503	42.481	0.183	0.875	372
Turkish & vaccinated	0.473	0.491	41.930	0.189	0.891	371
Turkish & unvaccinated	0.504	0.519	43.307	0.207	0.899	387
CDU & vaccinated	0.528	0.470	42.622	0.165	0.859	370
CDU & unvaccinated	0.505	0.454	42.887	0.174	0.880	390
SPD & vaccinated	0.434	0.539	42.542	0.166	0.885	380
SPD & unvaccinated	0.511	0.476	43.855	0.150	0.892	380
FDP & vaccinated	0.509	0.471	43.206	0.190	0.876	384
FDP & unvaccinated	0.495	0.508	43.321	0.148	0.902	386
Greens & vaccinated	0.493	0.508	42.907	0.164	0.864	378
Greens & unvaccinated	0.446	0.513	42.718	0.141	0.866	390
Left Party & vaccinated	0.487	0.489	43.347	0.142	0.897	380
Left Party & unvaccinated	0.469	0.496	43.995	0.172	0.870	383
AfD & vaccinated	0.497	0.463	43.106	0.176	0.913	376
AfD & unvaccinated	0.484	0.508	42.781	0.173	0.861	388

Table S5: Regression models

Treatment	Estimate	SE	CI_{low}	CI_{high}	N
Germans towards Germans	6.439	1.125	4.231	8.646	732
Germans towards Turks	3.326	1.090	1.185	5.466	743
Mainstream party voters towards AfD	−25.595	1.188	−27.929	−23.260	480
AfD voters towards mainstream parties	− 3.862	1.235	− 6.288	− 1.435	418
Vaccinated towards vaccinated	10.209	0.510	9.208	11.210	2616
Vaccinated towards unvaccinated	−16.112	0.521	−17.133	−15.092	2651
Unvaccinated towards unvaccinated	12.874	1.469	9.985	15.764	358
Unvaccinated towards vaccinated	− 6.364	1.414	− 9.146	− 3.582	346

Note: Each row represents a separate linear regression model that includes only the dependent variable for the respective treatment group. Estimates refer to the mean of the thermometer variable. CI refers to the 95% confidence intervals.

Figure S1: Predicted sympathy values with 95% confidence intervals by nationality, party and vaccination status (including control variables). Sympathy ranges from 50 (low) to +50 (high). For interpretation: “Mainstream party voters towards AfD” refers to the sympathy of voters of mainstream parties (CDU/CSU, SPD, Greens, and FDP) towards AfD supporters. “Vaccinated towards unvaccinated” represents the sympathy of the vaccinated towards unvaccinated persons.

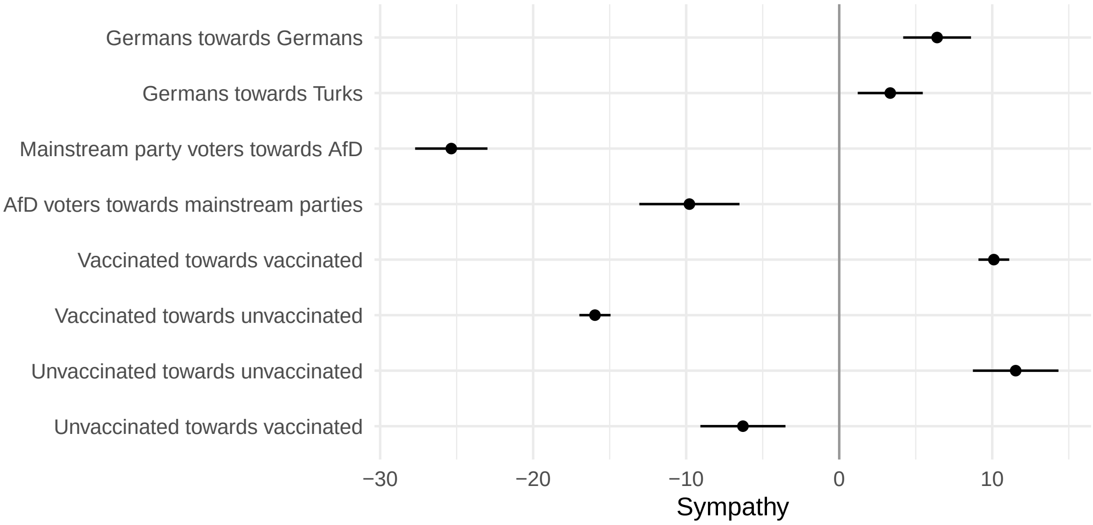

Table S6: Regression Models of Identification with Vaccination Status

	Vaccinated towards vaccinated	Vaccinated towards unvaccinated	Unvaccinated towards unvaccinated	Unvaccinated towards vaccinated
Intercept	-0.603 (2.682)	10.220 (2.665)***	-2.093 (7.510)	-2.958 (7.073)
	[$p = 0.822$]	[$p < 0.001$]	[$p = 0.781$]	[$p = 0.676$]
Vacc. status ID	3.170 (0.303)***	-3.745 (0.307)***	3.285 (0.791)***	-1.126 (0.817)
	[$p < 0.001$]	[$p < 0.001$]	[$p < 0.001$]	[$p = 0.169$]
East German	0.742 (1.434)	5.421 (1.450)***	0.868 (3.401)	0.478 (3.370)
	[$p = 0.605$]	[$p < 0.001$]	[$p = 0.799$]	[$p = 0.887$]
Education: high	0.107 (1.114)	-4.805 (1.117)***	-3.139 (3.164)	2.521 (3.239)
	[$p = 0.923$]	[$p < 0.001$]	[$p = 0.322$]	[$p = 0.437$]
Female	0.359 (1.100)	-1.055 (1.091)	6.862 (3.130)*	4.886 (3.084)
	[$p = 0.744$]	[$p = 0.334$]	[$p = 0.029$]	[$p = 0.114$]
Age	-0.092 (0.043)*	-0.178 (0.043)***	-0.086 (0.123)	-0.036 (0.125)
	[$p = 0.032$]	[$p < 0.001$]	[$p = 0.484$]	[$p = 0.773$]
R^2	0.043	0.077	0.070	0.017
Adj. R^2	0.042	0.075	0.056	0.001
N	2451	2501	332	323

Unstandardized estimates from linear regression models with standard errors in parentheses and p-values in square brackets. "Vacc. status ID" indicates strength of vaccination status. ***p<0.001; **p<0.01; *p<0.05.

C Additional Robustness Checks using Attention Checks and Removal of Speeders and Laggers

During the survey, a hypothetical interview was presented to the respondent (for a different purpose). The attention check consisted of a multiple selection list of eight items following the interview, of which three denote topics that have been discussed in the constructed expert interview (“Opportunities for advancement”, “Income inequality”, “Fair income”) and five denote topics that have not been discussed.

Passing the test in the *high threshold scenario* is defined as choosing all three correct items and choosing none of the incorrect items, with the additional condition of realistic latency times (an average of at least 4 seconds per interview page). Passing the test in the *low threshold scenario* is defined as choosing at least two of the three correct items, again with the additional condition of realistic latency times (an average of at least 4 seconds per interview page). The results are displayed in Figures S2–S5 and are basically identical to the findings presented in the main text.

Finally, we also tested whether the removal of speeders (fastest 3% of respondents, response time $<$ 343ms, n=175) and respondents with very long response times (response time $<$ 10,000ms, n=4) affects our results. The findings are presented in Figures S6 and S7 and are again quite similar to the results in the main text.

Figure S2: Predicted sympathy values with 95% confidence intervals by nationality and vaccination status using the attention check in the *low threshold scenario*. Sympathy ranges from 50 (low) to +50 (high). For interpretation: "Vaccinated towards unvaccinated" represents the sympathy of the vaccinated towards unvaccinated persons.

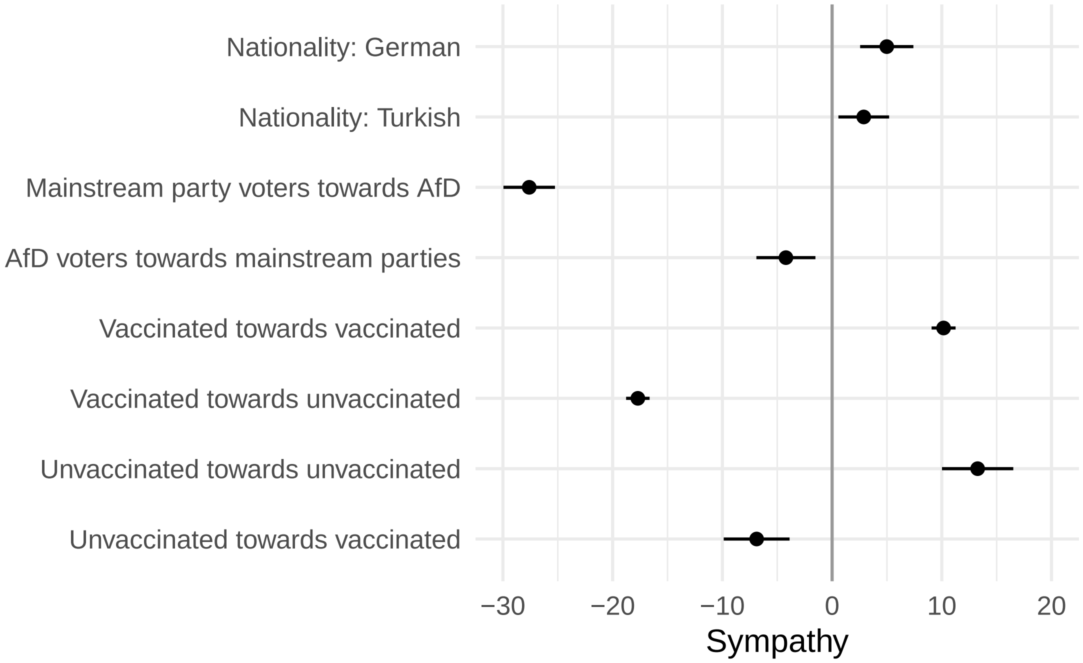

Figure S3: Predicted sympathy values with 95% confidence intervals by vaccination status identification using the attention check in the *low threshold scenario*. Sympathy ranges from 50 (low) to +50 (high), identification with vaccination status from 1 (none) to 7 (strong). For interpretation: “Vaccinated towards unvaccinated” represents the sympathy of the vaccinated towards unvaccinated persons.

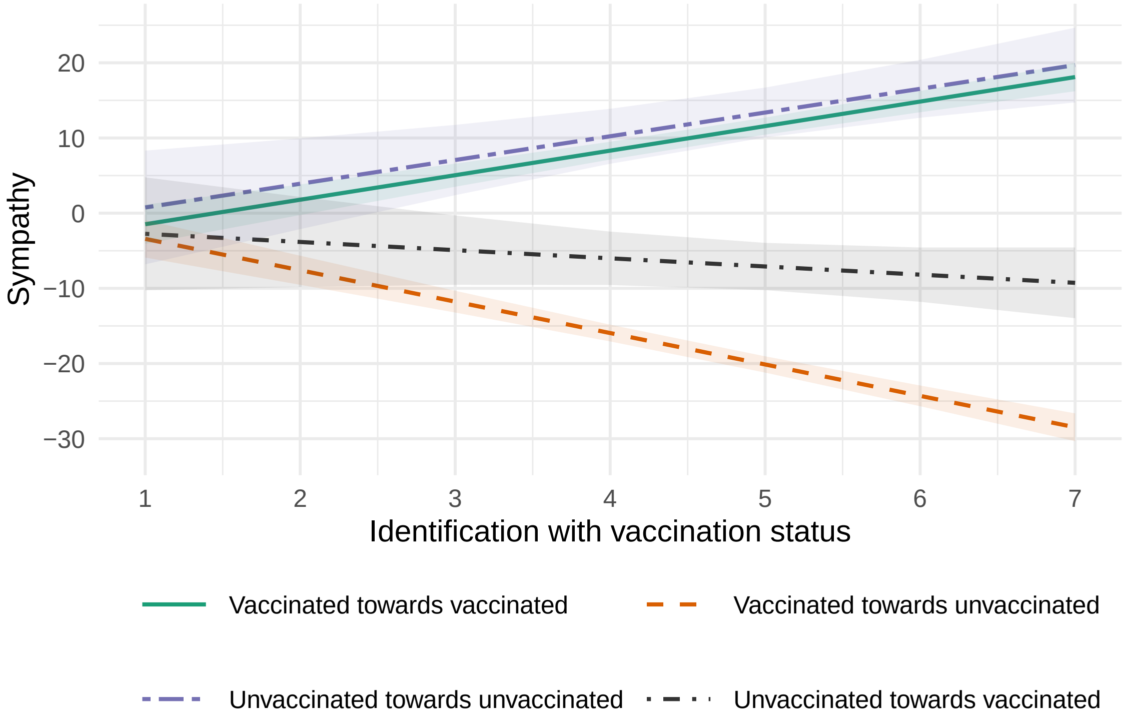

Figure S4: Predicted sympathy values with 95% confidence intervals by nationality and vaccination status using the attention check in the *high threshold scenario*. Sympathy ranges from 50 (low) to +50 (high). For interpretation: "Vaccinated towards unvaccinated" represents the sympathy of the vaccinated towards unvaccinated persons.

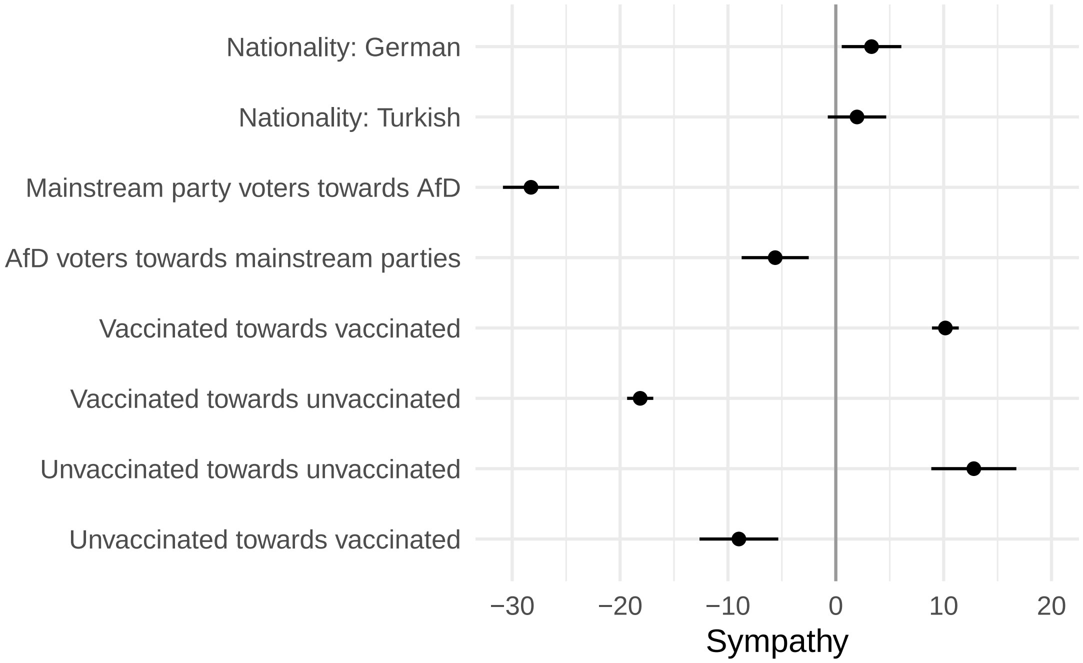

Figure S5: Predicted sympathy values with 95% confidence intervals by vaccination status identification using the attention check in the *high threshold scenario*. Sympathy ranges from 50 (low) to +50 (high), identification with vaccination status from 1 (none) to 7 (strong). For interpretation: "Vaccinated towards unvaccinated" represents the sympathy of the vaccinated towards unvaccinated persons.

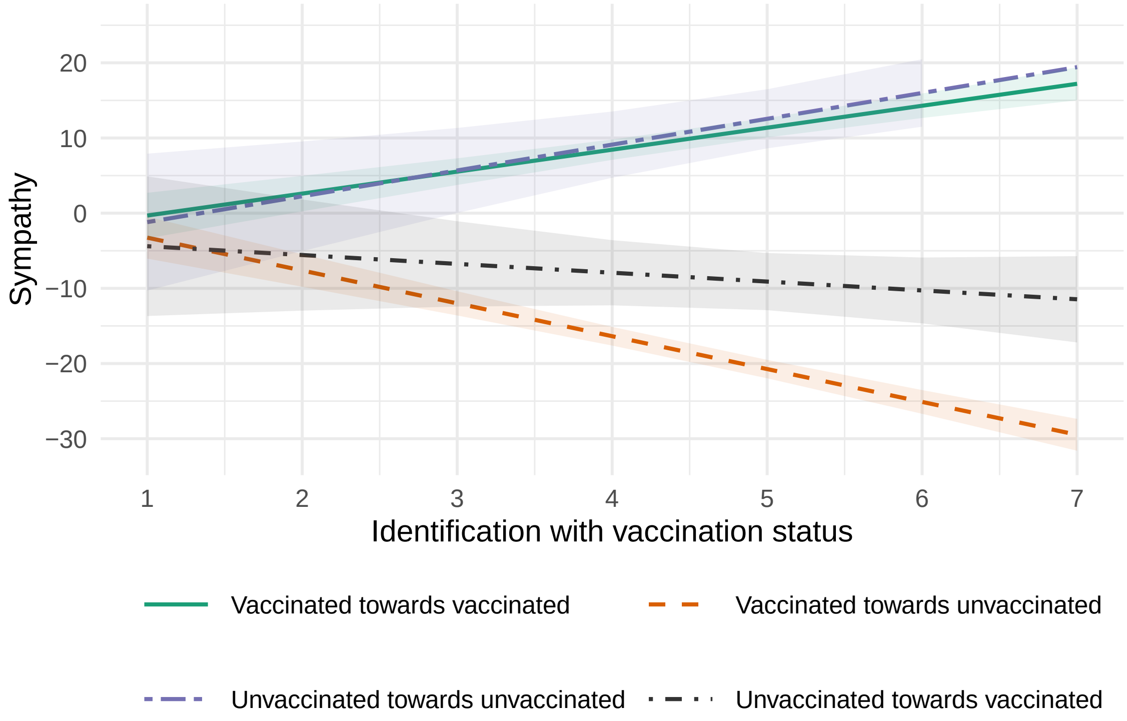

Figure S6: Predicted sympathy values with 95% confidence intervals by nationality and vaccination status (speeders and laggers removed). Sympathy ranges from 50 (low) to +50 (high). For interpretation: "Vaccinated towards unvaccinated" represents the sympathy of the vaccinated towards unvaccinated persons.

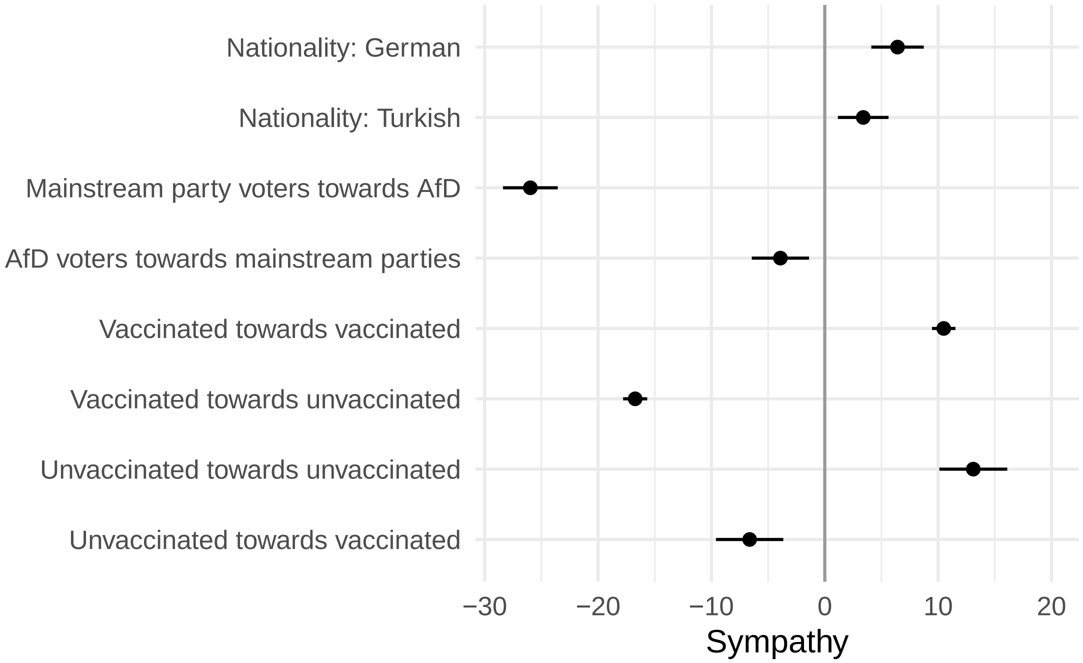

Figure S7: Predicted sympathy values with 95% confidence intervals by vaccination status identification (speeders and laggers removed). Sympathy ranges from 50 (low) to +50 (high), identification with vaccination status from 1 (none) to 7 (strong). For interpretation: “Vaccinated towards unvaccinated” represents the sympathy of the vaccinated towards unvaccinated persons.

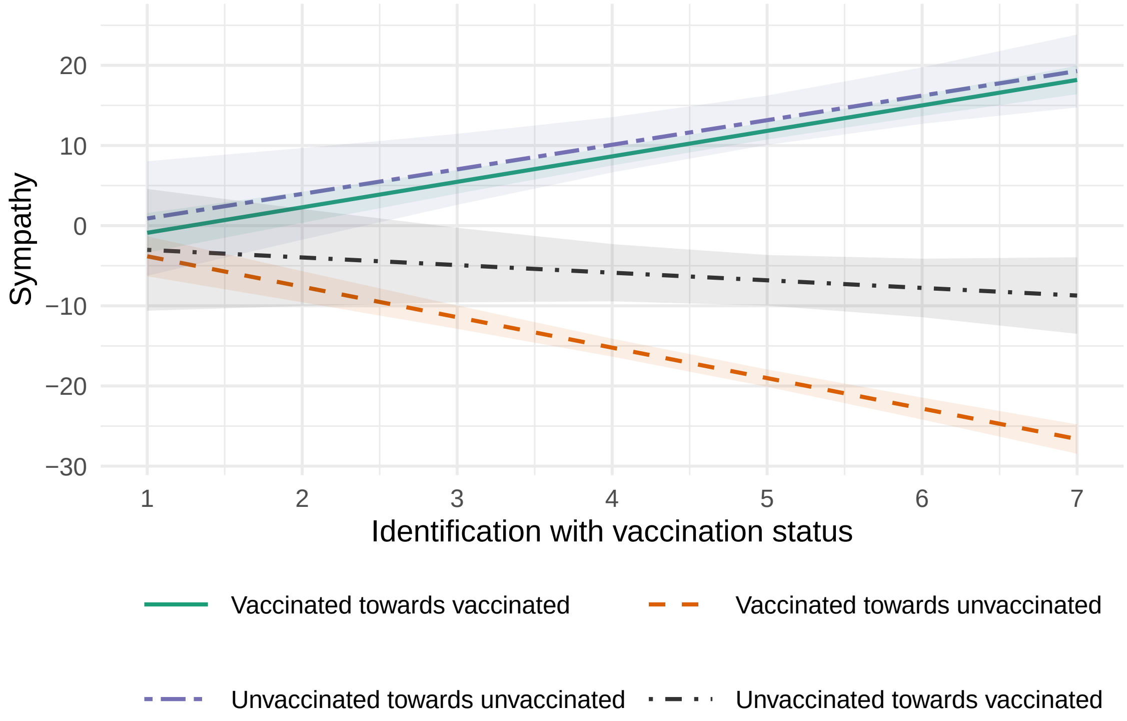

D Recommended Reporting Standards for Experiments according to the Experimental Standards Committee of the American Political Science Association

A) Hypotheses

- Specific objectives or hypotheses.
    - Hypotheses were stated in the Introduction of the manuscript.

B) Subjects and Context

- Eligibility and exclusion criteria for participants.
    - The survey was conducted by *respondi* (see `https://www.respondi.com/access-panel`). The company is ISO 26362 certified and the sample is representative according to age (18 to 65 years), education and sex (see Table S3). 85% of panelists were recruited through own campaigns with the rest being recruited through marketers and self-recruitment. The access panel in Germany consists of roughly 100.000 individuals with an average response rate of 40%. For our study, all members of the access panel aged 18–65 years were eligible to participate in the study. All participants provided informed consent at prior to

answering the questionnaire and they received a financial compensation (by *respondi*) after successfully completing the survey.

- The survey was conducted between December 10 and 19, 2021 (n=6,100). Respondents were presented with the vignette after a series of questions on age, sex, education, and political attitudes. All respondents were assigned a treatment, although four respondents did not answer the sympathy thermometer question.

C) Allocation Method

- Details of the procedure used to generate the assignment sequence (e.g., randomization procedures).
    - The treatment was assigned fully randomly across all individuals who participated in the study.
- If random assignment used, provide evidence of random assignment.
    - Table S4 provides evidence for random assignment of treatments according to age, sex, education and vaccination status. Age, sex and education were asked pre-treatment in all three surveys, whereas vaccination status was asked post-treatment.

D) Treatments

- Description of the interventions in each treatment condition, as well as a description of the control group.

        - See section A for a description of the vignette and treatment groups.

    - How and when manipulations or interventions were administered.

        - See section manuscript, section A, and preregistration for further explanation.

E) Results

1. Outcome Measures and Covariates.

    - Provide precise definition of all primary and secondary measures and covariates.

        - This is addressed in the manuscript and the preregistration.

    - Clearly state which of the outcomes and subgroup analyses were specified prior to the experiment and which were the result of exploratory analysis.

        - This is addressed in the manuscript, section A, and the preregistration. All analyses were preregistered.

2. Complete CONSORT Participant Flow Diagram.

    - This is addressed in the manuscript, section A, and the preregistration.

3. Statistical Analysis.

    - This is addressed in the manuscript and the preregistration. We did not weight our analyses.

F) Other

- Was the experiment reviewed and approved by an IRB?
    - No, not necessary. There was no ethics committee established at the author's institution at the time of the conception of the study. However, participants were part of an online access-panel of the survey company *respondi* (now *Bilendi&respondi*). The company is ISO 20252 certified (`https://www.iso.org/standard/73671.html`) and is bound to the guidelines by ESOMAR, irep, Adetem, and DGOF. See separate "Statement on ethics in conducting the survey".
- If the experimental protocol was registered, where and how can the filing be accessed?
    - The experimental protocol was preregistered at `https://aspredicted.org/blind.php?x=3BP_5SW`.
- What was the source of funding? What was the role of the funders in the analysis of the experiment?
    - This research is part of the project that has been funded. Due to anonymization this will be revealed after acceptance of the paper.
- If a replication data set is available, provide the URL.
    - I will upload the replication materials to the Harvard Dataverse after the paper has been accepted for publication.

References

Statistisches Bundesamt (Destatis), Wissenschaftszentrum Berlin für Sozialforschung (WZB), and Bundesinstitut für Bevölkerungsforschung (BiB) (2021). *Datenreport 2021. Ein Sozialbericht für die Bundesrepublik Deutschland*. Bonn: Bundeszentrale für politische Bildung.
